# Frequentist rules for regulatory approval of subgroups in phase III trials: A fresh look at an old problem

**DOI:** 10.1177/09622802211017574

**Published:** 2021-06-02

**Authors:** K Edgar, D Jackson, K Rhodes, T Duffy, C-F Burman, LD Sharples

**Affiliations:** 1 Department of Medical Statistics, London School of Hygiene and Tropical Medicine, London, UK; 2 Statistical Innovation, Oncology R&D, AstraZeneca, AstraZeneca, Cambridge, UK; 3 Wolfson Institute of Preventive Medicine, Queen Mary University of London, London, UK; 4 Statistical Innovation, BioPharmaceutical R&D, AstraZeneca, Gothenburg, Sweden

**Keywords:** Clinical trials, regulatory approval, subgroups, enrichment

## Abstract

**Background:**

The number of Phase III trials that include a biomarker in design and analysis has increased due to interest in personalised medicine. For genetic mutations and other predictive biomarkers, the trial sample comprises two subgroups, one of which, say B+ is known or suspected to achieve a larger treatment effect than the other B−. Despite treatment effect heterogeneity, trials often draw patients from both subgroups, since the lower responding B− subgroup may also gain benefit from the intervention. In this case, regulators/commissioners must decide what constitutes sufficient evidence to approve the drug in the B− population.

**Methods and Results:**

Assuming trial analysis can be completed using generalised linear models, we define and evaluate three frequentist decision rules for approval. For rule one, the significance of the average treatment effect in B− should exceed a pre-defined minimum value, say $Z_{B-}>L$. For rule two, the data from the low-responding group B− should increase statistical significance. For rule three, the subgroup-treatment interaction should be non-significant, using type I error chosen to ensure that estimated difference between the two subgroup effects is acceptable. Rules are evaluated based on conditional power, given that there is an overall significant treatment effect. We show how different rules perform according to the distribution of patients across the two subgroups and when analyses include additional (stratification) covariates in the analysis, thereby conferring correlation between subgroup effects.

**Conclusions:**

When additional conditions are required for approval of a new treatment in a lower response subgroup, easily applied rules based on minimum effect sizes and relaxed interaction tests are available. Choice of rule is influenced by the proportion of patients sampled from the two subgroups but less so by the correlation between subgroup effects.

## 1 Background

Since the rise of personalised medicine, the number of Phase III trials that include a biomarker in design and analysis has increased. A biomarker has been defined as “A … characteristic that is measured as an indicator of normal biological processes, pathogenic processes or responses to an exposure or intervention.”^1^ Biomarkers of interest are those which are related to important clinical outcomes and may be *prognostic* (associated with the clinical outcome independently of treatment) or *predictive* (interact with treatment).^2^ Predictive biomarkers in contemporary trials are often related to one or more genetic mutations, gene expression or a function of several genetic markers and may be dichotomous, ordinal or continuous. Despite the loss of information, for practical reasons they are often dichotomised. In this paper, we focus on biomarkers that are dichotomous (naturally or by design), and prior to commencing a confirmatory Phase III trial, are expected to be predictive.

In our context, we define sub-populations of patients as either biomarker positive (B+) or negative (B−). A common situation is that the treatment efficacy is *a priori* assumed to be better (or at least as good) in B+ compared to B− patients. For example,

1. a drug treatment may have been developed to target a genetic disorder defining group B+,
2. there may be an untested clinical hypothesis of better efficacy in B+,
3. empirical data (biological or early clinical) may indicate higher efficacy in B+.

Even if a treatment has been developed to target the biomarker of interest, some of the efficacy in B+ may obtain in B−. Depending on the situation, one may expect no or minimal efficacy in B−, that a large proportion of the efficacy in B+ is retained, or that efficacy in B− is difficult to predict even if the treatment is known to be efficacious in B+. The effect in B− may arise, for example, if the biomarker is intrinsically continuous, so that treatment efficacy varies continuously across its levels. Dichotomisation may then lead to positive, but smaller efficacy in B−. Alternatively, some patients in B− may have an unknown genetic defect acting on the same pharmacological pathway as the B+ genetic mutation, which is the target of the intervention. Moreover, few biomarker tests have 100% sensitivity and specificity in practice, leading to diffusion of efficacy from patients incorrectly classified as B+ or B−. As a result, biomarker level will interact with the treatment, with a higher treatment effect in B+ patients than B− patients.

Although a new treatment may be most effective in the higher responding B+ subgroup, trials often draw patients from both subgroups, since the lower responding B− subgroup may also gain benefit from the intervention. In this case, a problem emerges when deciding what constitutes sufficient evidence to approve the drug in the B− population. European guidelines state that “Confirmatory trials should reflect the target population to be treated” so that trials will sample from a target population. However, if the target population is heterogeneous, ensuring that the treatment effect is sufficiently large in a lower responding subgroup may still be warranted. Moreover, in order to improve efficiency of a trial, the higher responding subgroup may be oversampled (enriched sample), resulting in under-representation of lower responders relative to the target population. In such a case, regulators and sponsors are concerned that automatic approval for lower responders based on obtaining an overall significant effect could result in harm, for example if side effects outweigh potential benefit in this subgroup. If the low response B− subgroup makes up a very large proportion in the population, there may also be substantial cost to healthcare providers, but not the predicted benefits. Therefore, regulators and sponsors may wish to impose additional conditions for approval of the treatment in this subgroup. Current regulatory guidelines acknowledge the importance of heterogeneity in decision making and encourage subgroup analysis in confirmatory trials.^3^ However, the guidance does not describe specific rules for approval of subgroups when heterogeneity exists.

There is a large literature on subgroup analysis in phase III trials. Much of this literature concerns *post hoc* exploration of a moderate to large number of subgroups using interaction tests, with issues such as data dredging and multiplicity well documented.^4^ This study differs in that we are concerned with the situation where there is an overall significant effect, two pre-defined subgroups known to differ in treatment effect, and optimal rules for treatment approval in the lower responding subgroup are required.

Where hypothesis tests are applied to multiple subgroups, it is important to control family-wise error rate (FWER).^5^ For two subpopulations B+ and B−, there are different multiple testing procedures that control the FWER.^6^ In most applications, formal testing focuses on F and B+ using either a hierarchical approach (F followed by B+, or B+ followed by F) or by splitting type I error between parallel tests; testing of B− is rarely included in applications for regulatory approval, as power is considered to be limited.^7,8^ In this study, we concentrate on the situation where the intervention has statistically significant efficacy in F, with significance in the B+ group assumed to follow due to higher efficacy in this subpopulation; conditions for approval in B−are then developed and assessed. A strategy that conditions on significance in B+ rather than F is closely related mathematically and results are expected to be very similar.

Although the study is motivated by trials of drugs targeting specific genetic mutations (see Gonzalez-Martin et al.^9^ for a recent example), other examples of trials with similar structures define subgroups according to age (adults and children),^10^ mild and severe disease,^11^ early and late stage cancers,^12^ as well as other biomarkers.^13^ Proposed methods should apply to any trial including two subgroups with known or suspected treatment effect inequality.

We provide a brief literature review of methods and practice in this context in section 2 before describing proposed rules for exponential family models in section 3. Conditional power of the rules is explored in section 4 and applied retrospectively to two published phase III trials in section 5, before briefly discussing implications for future trial design. A discussion completes the paper (section 6).

## 2 Existing literature

A review of FDA drug approvals with required biomarker testing found that biomarker negative patients were simply excluded from the majority of trials.^14^ Since exclusion was often not based on clinical evidence, these patients could be denied potential benefit from novel treatments. Moreover, provided that there is a sound biological basis for some benefit in biomarker negative patients, including them may also confirm the clinical utility of the biomarker itself.

Heterogeneity within a target population was also recognised in updated EMA guidance on the investigation of subgroups in confirmatory clinical trials, published in January 2019.^3^ Whilst the guidance suggests that restriction of a trial population to a sub-population is justified if there are safety concerns or an anticipated lack of efficacy, it also calls for additional trials including the full breadth of the population to provide the best evidence of effect modifiers. Inclusion of the biomarker negative subgroup was highlighted as important, though it can create difficulties in analysis if the treatment effect is small or there are only a small number of such patients, resulting in low power to detect a significant treatment effect. Despite this, patients in this subgroup may still benefit from treatment, and are therefore harmed if the result is discarded for non-significance.

A 2016 review by Ondra et al.^15^ found that two concepts underpin current methods for assessing subgroup effects, influence and interaction. An ‘influence’ condition sets a threshold that must be met by the treatment estimate of the subgroup of interest, whilst an interaction test sets a difference between treatment effects for two (or more) subgroups, in effect requiring that effects are sufficiently close. These methods may be used for approval of a treatment or as conditions which must be met for the subpopulation to be included in the next stage of analysis (adaptive designs). For example, Stallard et al.^16^ compared different strategies for choosing which hypotheses to test in the second stage of analysis (either the full population or a subgroup, or both), which used either an influence or interaction test approach. Similarly, Matsui and Crowley^17^ proposed a sequential design where in the first stage of their analysis they use superiority and futility boundaries to decide which populations go forward for further analysis. This preserves statistical power for detecting various profiles of treatment effects across the subgroups, and allows the biomarker negative population to be tested again if they do not cross the futility boundary.

Despite the development of different adaptive designs, interaction tests appear to be the main method used to assess subgroup heterogeneity. In our (unpublished) targeted systematic review of large clinical trials that carried out subgroup analyses in the *New England Journal of Medicine*, we found that approximately two thirds used interaction tests to decide whether there was significant treatment effect heterogeneity. Almost all other articles summarised within-subgroup effects and used significance tests with 5% type I error.

Although most of the literature rests on the frequentist paradigm, a Bayesian approach could also be considered.^18^ By specifying a two-dimensional prior for efficacy in B+ and B−, one can explicitly borrow information from one subpopulation when evaluating the other. This prior should reflect the clinical plausibility of a range of differential treatment effects between the two subpopulations.

This study was partly motivated by the design of the APEX trial, which compared betrixaban with standard dose enoxaparin in medically ill patients at risk of venous thrombosis. They carried out sequential analyses, the first on a subgroup defined by the biomarker D-dimer, the second on a subgroup defined by a combination of D-dimer level and age, and the third of the full population. If any result was negative, then subsequent tests were treated as exploratory. The first subgroup analysis was just above the pre-defined threshold for statistical significance of 5% (*p* = 0.054), so that the subsequent subgroup analysis (elevated D-dimer level and age ≥75) and full population analysis had to be treated as exploratory, although hypothesis test statistics were ‘significant’ at *p* = 0.03 and *p* = 0.006, respectively. Clearly, such an analysis may have substantial implications for approval of the experimental treatment. A more traditional analysis plan would be to consider the full population first followed by the subgroups; however, conditions for approval in subgroups are less well established.

In our context, we may accept some heterogeneity between subgroups, provided that there is sufficient benefit in the B− subgroup. The issue is in choosing an acceptable difference between the treatment effect in the two subgroups, or equivalently, choosing a relaxed (higher) significance level for the interaction test. On the other hand, decision rules that focus on influence rely solely on the data in the B− subgroup, but require us to pre-specify a minimum bound for the acceptance threshold. In order to avoid the need to specify either a minimum treatment effect or a more relaxed interaction level, a decision rule that does not require additional parameters may also be attractive.

The question of how to deal with approval in a limited sub-population has important implications for maximising the patients who could benefit, which is particularly important for conditions where there are few treatment options. There remains uncertainty about how to address this issue, and how different decision rules perform according to issues such as prevalence of the high responder subgroup in the population and in the trial. We outline a simple strategy to choose appropriate and efficient decision rules in a frequentist framework.

## 3 Methods

### 3.1 Generalised linear model and subgroup notation

In practice, phase III clinical trials that have a biomarker-treatment interaction are analysed using linear, generalised linear or survival regression models. We restrict attention in this paper to the wide range of trial outcomes that have Normal, Binomial or Poisson distributions and review the general framework here, defining estimands of interest, estimators and statistics.

Generalised linear models that describe different treatment effects in the two subgroups have a linear predictor of the form

(1) $$\eta_{i}=\beta_{0}+\beta_{1}T_{i}+\beta_{2}S_{i}+\beta_{3}(T_{i}\times S_{i})+\beta_{4}^{T}X_{i}$$

where for patient $i,i=1,\ldots ,N,\;T_{i}=0,1$ for control and experimental treatments, $S_{i}=0,1$ for subgroups B− (biomarker negative), B+ (biomarker positive) and $X_{i}$ is a vector of baseline covariates, usually minimisation or stratification factors, included to increase precision of the treatment effect estimate or to adjust for chance imbalance. We assume that there are *N* patients in the trial overall, n=N/2 in each trial arm and that *πn* in each treatment arm are drawn from sub-population B+, the remaining (1−π)n are drawn from sub-population B−.

For the exponential family of distributions, the expected response and the linear predictor are connected through the link function $g(\gamma_{i})=\eta_{i}$. For trial outcomes that have Normal, Binomial or Poisson distributions, canonical link functions are the identity, logit and log functions, respectively.

We define the **estimands of interest** in the two sub-populations as the treatment effects $\mu_{B+}=\beta_{1}+\beta_{3}$ for the biomarker positive subgroup and $\mu_{B-}=\beta_{1}$ for the biomarker negative subgroup. Without loss of generality, positive values of $\mu_{B+}$ and $\mu_{B-}$ indicate that the treatment is beneficial. For Normal response variables, these are mean treatment effects in the two subgroups, for Binomial responses they are log odds-ratios and for Poisson responses they are log rate-ratios. **Estimators** of these estimands can be obtained using maximum likelihood as ${\hat{\mu }}_{B+}={\hat{\beta }}_{1}+{\hat{\beta }}_{3}$ and ${\hat{\mu }}_{B-}={\hat{\beta }}_{1}$. We can also estimate the approximate maximum likelihood (co-)variance components from the information matrix, so that for generalised linear models

$$\left(\begin{array}{c} {\hat{\beta }}_{1}+{\hat{\beta }}_{3}\\ {\hat{\beta }}_{1} \end{array}\right)∼\mathrm{BVN}\left(\left(\begin{array}{c} \beta_{1}+\beta_{3}\\ \beta_{1} \end{array}\right),\left(\begin{array}{cc} \mathrm{Var}({\hat{\beta }}_{1})+\mathrm{Var}({\hat{\beta }}_{3})+2Cov({\hat{\beta }}_{1},{\hat{\beta }}_{3}) & \mathrm{Var}({\hat{\beta }}_{1})+\mathrm{Cov}({\hat{\beta }}_{1},{\hat{\beta }}_{3})\\ \mathrm{Var}({\hat{\beta }}_{1})+\mathrm{Cov}({\hat{\beta }}_{1},{\hat{\beta }}_{3}) & \mathrm{Var}({\hat{\beta }}_{1}) \end{array}\right)\right)$$

For inference for each group separately, we define *Z*-statistics

$Z_{B+}={\hat{\mu }}_{B+}/{\hat{\sigma }}_{B+}$ and $Z_{B-}={\hat{\mu }}_{B-}/{\hat{\sigma }}_{B-}$where ${\hat{\sigma }}_{B+}$ and ${\hat{\sigma }}_{B-}$ are estimates of the standard errors of the estimands taken from the information matrix.

### *3.2 Likelihood assuming no correlation between* $\mu_{B+}$ *and* $\mu_{B-}$

In order to gain insight into the contribution of *π*, the proportion of trial patients drawn from the high-responding subgroup B+, it is useful to consider the case where the trial analysis is not adjusted for baseline factors, so that there is zero correlation between $\mu_{B+}$ and $\mu_{B-}$. In this case we can write

$${\hat{\beta }}_{1}+{\hat{\beta }}_{3}\equiv {\hat{\mu }}_{B+}∼N(\mu_{B+},\sigma_{B+}^{2})$$

and independently

$${\hat{\beta }}_{1}\equiv {\hat{\mu }}_{B-}∼N(\mu_{B-},\sigma_{B-}^{2})$$

Specifically, for the response *Y_i_*, i=1,…,N, with canonical link and assuming 1:1 randomisation between treatment arms, the variance components can be approximated by:

1. Normal distribution-Identity link $\sigma_{B+}^{2}=2\sigma^{2}/\pi n$ and $\sigma_{B-}^{2}=2\sigma^{2}/(1-\pi )n$, where $\sigma^{2}$ is the sampling variance.
2. Binomial distribution-Logit link $\sigma_{B+}^{2}=[1/(\theta_{1B+}(1-\theta_{1B+}))+1/(\theta_{0B+}(1-\theta_{0B+})]/\pi n$ and $\sigma_{B-}^{2}=[1/(\theta_{1B-}(1-\theta_{1B-}))+1/(\theta_{0B-}(1-\theta_{0B-})]/((1-\pi )n)$ where *θ_jk_* is the probability of an event in treatment arm *j* and subgroup *k.*
3. Poisson distribution-Log link $\sigma_{B+}^{2}=[1/\lambda_{1B+}+1/\lambda_{0B+}]/\pi n$ and $\sigma_{B-}^{2}=[1/\lambda_{1B-}+1/\lambda_{0B-}]/((1-\pi )n)$ where *λ_jk_* is the event rate in treatment arm *j* and subgroup *k*.

We note that in all three cases the variance includes the term 1/π or 1/(1−π), which will facilitate investigation of the influence of the distribution of the sub-populations in the trial.

#### 3.2.1 Making inferences in the full population in the general case.

We write the full population treatment effect as

(2) $$\mu_{F}=\pi \mu_{B+}+(1-\pi )\mu_{B-}$$

That is, the estimand for the full population is a weighted average of the subgroup specific estimands $\mu_{B+}$ and $\mu_{B-}$, with weights given by the proportion of the trial sample drawn from each sub-population, *π* and 1−π. Note that, for *μ_F_* to be directly interpretable, these proportions should hold in the target population, otherwise some translation is required.

Since $Z_{B+}={\hat{\mu }}_{B+}/\sigma_{B+}$ and $Z_{B-}={\hat{\mu }}_{B-}/\sigma_{B-}$, we have

$${\hat{\mu }}_{F}=\pi {\hat{\mu }}_{B+}+(1-\pi ){\hat{\mu }}_{B-}=\pi \sigma_{B+}Z_{B+}+(1-\pi )\sigma_{B-}Z_{B-}$$

If *ρ* is the correlation between the estimands, the *Z*-statistic for the full population is

(3) $$Z_{F}=\frac{{\hat{\mu }}_{F}}{\sigma_{F}}=\frac{\pi \sigma_{B+}Z_{B+}+(1-\pi )\sigma_{B-}Z_{B-}}{\sqrt{\pi^{2}\sigma_{B+}^{2}+{\left(1-\pi \right)}^{2}\sigma_{B-}^{2}+2\pi (1-\pi )\rho \sigma_{B+}\sigma_{B-}}}$$

If there are no additional covariates in the model ($\beta_{4}\equiv 0$), then $\mathrm{Cov}({\hat{\beta }}_{1},{\hat{\beta }}_{3})\equiv -\mathrm{Var}({\hat{\beta }}_{1})$, so that $\mathrm{Cov}({\hat{\beta }}_{1},{\hat{\beta }}_{1}+{\hat{\beta }}_{3})\equiv 0$ and the correlation *ρ* is zero.

Alternatively, if $\beta_{4}\neq 0$ then $\mathrm{Cov}({\hat{\beta }}_{1},{\hat{\beta }}_{1}+{\hat{\beta }}_{3})\neq 0$, and the correlation *ρ* describes the association between estimated effects due to adjustment for covariates. In general, the correlation induced by covariance adjustment is expected to be small.

As an aside, we note that the correlation between the statistics $Z_{B+}$ and $Z_{B-}$ is also equal to *ρ*.

### *3.3 Proposed rules for approval of the drug in* B−

### 3.3.1 Sequential testing and conditional power.

We define and evaluate three proposed rules for approval in the lower response population B− conditional on significance in the full population. Recall that we expect the treatment to be as effective or less effective in the B− population, but nevertheless it may be sufficiently effective to warrant approval. In this situation, evaluation of B− is only worthwhile if a significant effect has been established in the full population. Thus, we adopt a sequential testing strategy, first evaluating treatment in the full population and, conditional on a significant result, evaluating the treatment in B−. The conditional power of a decision rule is a natural method for assessing its value in this context; for decision rule *Rn*, conditional power is defined as

(4) $$P(Rn|Z_{F}>1.96)=\frac{P(Rn,Z_{F}>1.96)}{P(Z_{F}>1.96)}$$

(As an aside, for binary data where the analysis adjustment for baseline covariates is not required, this conditional probability can be calculated in closed form.)

The denominator does not depend on the form of any proposed rule and is given by the observed statistic in the full population

(5) $$P(Z_{F}>1.96)=\Phi (\mu_{F}/\sigma_{F}-1.96)$$

Note that the right-hand side is a function of three location parameters $\mu_{B+},\;\mu_{B-}$ and *π*, and three variance parameters $\sigma_{B+},\;\sigma_{B-}$ and *ρ* (see equations (2) and (3)), and conditional on these the standard normal deviate can be obtained from any statistical software.

We now consider conditional power of three classes of decision rule for approval of the drug in B−, summarised in Table 1.

**Table 1.** Proposed rules for approval in the lower treatment response subgroup B−.

Rule	Condition	Explanation
1	$Z_{B-}>L$	The *Z*-statistic in B− must exceed a pre-defined threshold *L*
		
2	$Z_{F}>Z_{B+}$	Results from B− must increase overall significance
3	$\frac{{\hat{\mu }}_{B+}-{\hat{\mu }}_{B-}}{SD({\hat{\mu }}_{B+}-{\hat{\mu }}_{B-})}<Z_{\alpha_{I}/2}$	No significant subgroup-treatment interaction at *α_I_* level

Rules 1 and 2 are different types of influence rule, whilst Rule 3 is an interaction test. The algebra for calculating the conditional power of each rule is given in full in Appendix 1.

### *3.4 Rule 1: the statistic*$Z_{B-}$*exceeds a pre-defined threshold* L

For the treatment to be acceptable in the B− subgroup, the significance of the average treatment effect should exceed a pre-defined minimum value, say $Z_{B-}>L$. Given prior estimates of $\mu_{B-}$ and its standard deviation, we could calculate the sample size to ensure this threshold is achieved with a given probability. As an absolute minimum, the treatment effect and associated statistic should be positive (*L *>* *0, assuming without loss of generality that positive effects signify treatment benefit), although such a mild condition is unlikely to be acceptable unless the drug has negligible adverse effects and little cost. Alternatively, setting *L *>* *1.96 is a strict condition requiring a significant treatment effect in the B− subgroup at traditionally accepted levels. This is tantamount to repeating the trial in the B− sub-population and will not be feasible if either this sub-population is small or difficult to recruit from, or the treatment effect is modest. Despite this, for serious diseases with no alternative effective treatments, a smaller expected treatment effect may be sufficient to outweigh any concerns regarding safety and cost; in this case a value L∈(0,1.96) should be pre-defined. In general, we may accept an intermediate value for *L*.

For rule 1, conditional power is given by the expression

$$P(Z_{B-}>L|Z_{F}>1.96)=\frac{P(Z_{B-}>L,Z_{F}>1.96)}{P(Z_{F}>1.96)}$$

where the denominator is defined in equation (5) and can be obtained from standard statistical software.

For the numerator $P(Z_{B-}>L,Z_{F}>1.96)$, writing $X=1.96-Z_{F}$ and $Y=L-Z_{B-}$ we show in Appendix 1 that the joint distribution of (X,Y) is

$$\left(\begin{array}{c} X\\ Y \end{array}\right)∼\mathrm{BVN}\left(\left(\begin{array}{c} 1.96-\mu_{F}/\sigma_{F}\\ L-\mu_{B-}/\sigma_{B-} \end{array}\right),\left(\begin{array}{cc} 1 & \frac{\pi \rho \sigma_{B+}+(1-\pi )\sigma_{B-}}{\sigma_{F}}\\ \frac{\pi \rho \sigma_{B+}+(1-\pi )\sigma_{B-}}{\sigma_{F}} & 1 \end{array}\right)\right)$$

Again, the numerator of the conditional power is a function of three location and three variance parameters $\mu_{B+},\;\mu_{B-}$, *π*, $\sigma_{B+},\;\sigma_{B-}$, *ρ*, through *μ_F_* and *σ_F_*. Conditional on these, the numerator is P(X≤0,Y≤0) and can be obtained from any statistical software.

### *3.5 Rule 2: The* B− *data should increase statistical significance*

For interventions with a low adverse event profile, approval may be acceptable provided that the data in B− are not in conflict with those in B+. More formally, we might approve in B− provided that the data increase statistical significance, that is, on condition that $Z_{F}>Z_{B+}$. For rule 2 the conditional power has denominator defined in equation (5) and numerator given by $P(Z_{F}>Z_{B+},Z_{F}>1.96)$.

Making the transformations, $X=1.96-Z_{F}$ and $Y=Z_{B+}-Z_{F}$, we show in Appendix 1 that the joint distribution of X and Y is

$$\left(\begin{array}{c} X\\ Y \end{array}\right)∼\mathrm{BVN}\left(\left(\begin{array}{c} 1.96-\mu_{F}/\sigma_{F}\\ \mu_{B+}/\sigma_{B+}-\mu_{F}/\sigma_{F} \end{array}\right),\left(\begin{array}{cc} 1 & 1-\frac{\pi \sigma_{B+}+\rho (1-\pi )\sigma_{B-}}{\sigma_{F}}\\ 1-\frac{\pi \sigma_{B+}+\rho (1-\pi )\sigma_{B-}}{\sigma_{F}} & 2\times (1-\frac{\pi \sigma_{B+}+\rho (1-\pi )\sigma_{B-}}{\sigma_{F}}) \end{array}\right)\right)$$

For the conditional power numerator, we calculate P(X≤0,Y≤0) using standard statistical software, conditional on subgroup-specific parameters.

### 3.6 Rule 3: No significant subgroup-treatment interaction at α_I_ level

When a range of subgroup effects are explored (often post hoc), it is customary to perform interaction tests to identify specific subgroups for which the treatment appears particularly effective/ineffective for further investigation. From our targeted systematic review of literature, the type I error rate *α_I_* is almost invariably set to 5%, with no adjustment for multiplicity. Our objective here is quite different; specifically we use *α_I_* as a measure of how confident we are that the two subgroups have different treatment effects, in order to decide whether approval in B− is warranted. In this case, we might choose a value for *α_I_* that is greater than 5%, depending on our knowledge of the variation of the treatment effects and the number of trial participants in each of the four subgroup-treatment combinations.

For this rule, the numerator is $P(({\hat{\mu }}_{B+}-{\hat{\mu }}_{B-})/SD({\hat{\mu }}_{B+}-{\hat{\mu }}_{B-})<z_{\alpha_{I}/2},Z_{F}>1.96)$, where $z_{\alpha_{I}/2}$ is the $100(1-\alpha_{I})\%$ quantile from the standard normal distribution and *SD* is the standard error of the difference in treatment effects in the two subgroups. We make the transformations, $X=1.96-Z_{F}$ and $Y=\frac{{\hat{\mu }}_{B+}-{\hat{\mu }}_{B-}}{SD({\hat{\mu }}_{B+}-{\hat{\mu }}_{B-})}-z_{\alpha_{I}/2}$, and write $SD({\hat{\mu }}_{B+}-{\hat{\mu }}_{B-})=\sigma_{3}=\sqrt{\sigma_{B+}^{2}+\sigma_{B-}^{2}-2\rho \sigma_{B+}\sigma_{B-}}$. Then we show in Appendix 1 that the joint distribution of X and Y is

$$\left(\begin{array}{c} X\\ Y \end{array}\right)∼\mathrm{BVN}\left(\left(\begin{array}{c} 1.96-\mu_{F}/\sigma_{F}\\ \frac{\mu_{B+}-\mu_{B-}}{\sigma_{3}}-z_{\alpha_{I}/2} \end{array}\right),\left(\begin{array}{cc} 1 & \frac{\rho (2\pi -1)\sigma_{B+}\sigma_{B-}-\pi \sigma_{B+}^{2}+(1-\pi )\sigma_{B-}^{2}}{\sigma_{F}\sigma_{3}}\\ \frac{\rho (2\pi -1)\sigma_{B+}\sigma_{B-}-\pi \sigma_{B+}^{2}+(1-\pi )\sigma_{B-}^{2}}{\sigma_{F}\sigma_{3}} & 1 \end{array}\right)\right)$$

Again, the numerator for conditional power is P(X≤0,Y≤0), which we obtain from standard statistical software, conditional on subgroup-specific parameters.

### 3.7 Illustration of proposed rules

**Figure 1.**
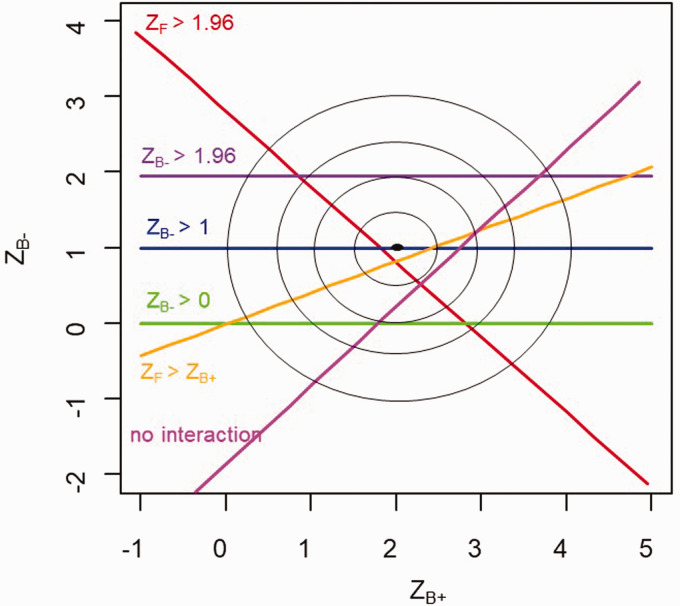
Illustration of the proposed rules in the $Z_{B+},Z_{B-}$ plane: regions above the lines are where Rules are met.

Figure 1 illustrates the proposed rules for the (hypothetical) case of independent normally distributed estimands for the two subgroups (on the scale of analysis e.g. linear, log, logistic). The statistics $Z_{B+}$ and $Z_{B-}$ have a bivariate normal distribution with mean (2, 1) and variance diag(1), represented by the unlabelled contours. The condition that the hypothesis test is significant in the full population is represented by the volume under the ($Z_{B+},Z_{B-}$) joint density that lies above and to the right of the red line. The volumes under the ($Z_{B+},Z_{B-}$) joint density that lie above the purple, blue and green lines represent Rule 1 for the cases where *L *=* *1.96 (treatment effect in B− is significant), *L *=* *1 (treatment effect in B− is one standard error) and *L *=* *0 (treatment effect is positive), respectively. The volume that lies above the orange line represents Rule 2 (B− results add to the overall significance) and the volume that lies above the pink line represents Rule 3 (interaction not significant at the one-sided 10% significance level). Conditional power for a certain rule, e.g. $P(Z_{B-}>1|Z_{F}>1.96)$, is the proportion of the total probability above the red curve ($Z_{F}>1.96$), that also lies above the boundary defined by the rule (e.g. above the blue line, so that $Z_{B-}>1$).

**Figure 2.**
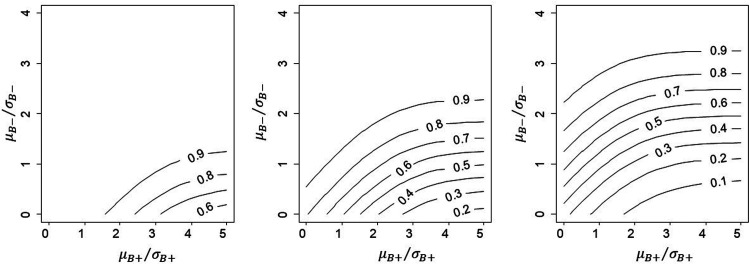
Illustration of conditional power for the case where ρ=0 and π=0.5 and for Rule 1: L = 0, 1, 1.96 (i.e. statistically significant).

In general, the conditional power for each of these three rules is given by the proportion of the density of (*X*, *Y*) that is consistent with the condition $Z_{F}>1.96$. As a specific example, consider Rule 1. Figure 2 shows the conditional power for Rule 1 for the case where estimands for B+ and B− are independent (*ρ* = 0), participants are drawn in equal numbers from the two sub-populations (π=(1−π)=0.5) and thresholds for approval set at L=0,1,1.96.

Because $Z_{B+}$ enters into the power calculation only through $X=1.96-Z_{F}$, it is independent of the approval threshold *L*, so that the *Y*-axis does not change position for different values of *L*. In contrast, as *L* increases, the joint density of (*X*, *Y*) shifts down the *Y*-axis and the proportion above zero decreases, thus decreasing the conditional power as expected. As the two estimands are assumed independent, the contours are circular. From the covariance matrix for Rule 1, *X* and *Y* will be positively correlated if the two estimands $\mu_{B+}$ and $\mu_{B-}$ are (ρ>0) and vice versa.

Similar patterns can be found for Rules 2 and 3.

## 4 Results

### 4.1 Comparison of conditional power for the proposed rules

The conditional power for all three rules depends on the relative treatment effects in the two subgroups, which is driven by the biological mechanisms of the treatment. Above this, we explore how the proportion sampled from each sub-population *π* and 1−π and the correlation between estimands *ρ* affects the power of the proposed rules.

### *4.2 Comparison of conditional power for proposed rules when* $\sigma_{B+}=\sigma_{B-},\;\pi =0.5$ *and **ρ** = 0*

**Figure 3.**
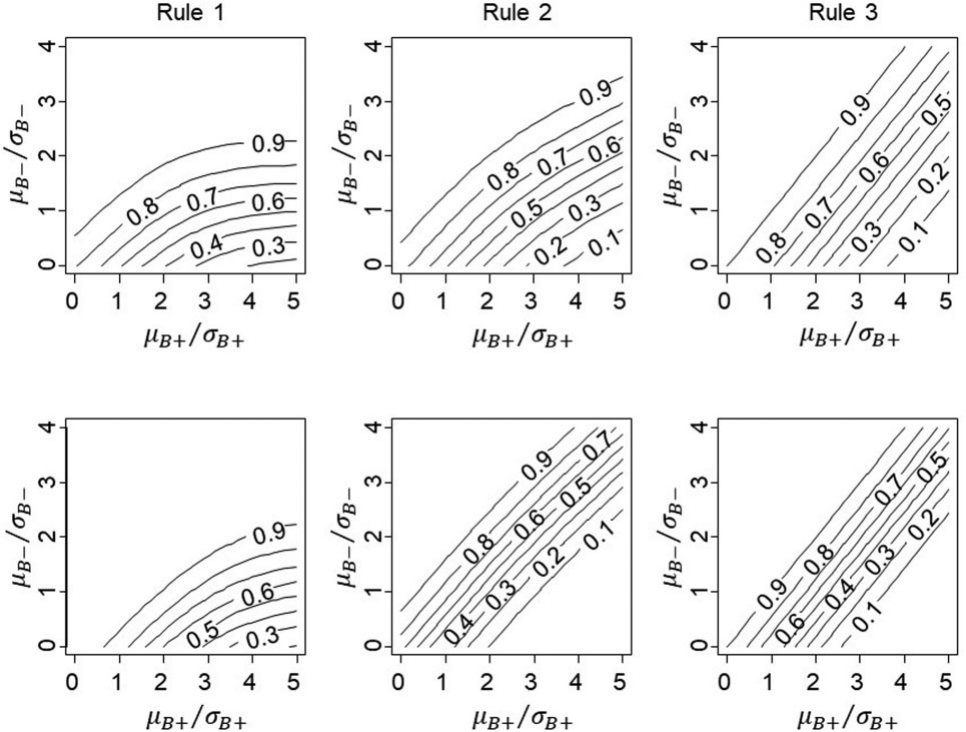
Conditional power for proposed rules with correlation between $\mu_{B-}$ and $\mu_{B+}$ equal to zero (top row) and ρ=0.5 (bottom row), with *L *=* *1 for Rule 1 and one-sided significance of 10% for Rule 3.

The top row of Figure 3 shows the relationship between the subgroup-specific statistics for treatment effect and conditional power in the simple case of equal standard errors, equal numbers in the two subgroups and independent estimands. For any value of $\mu_{B-}/\sigma_{B-}$, power decreases as $\mu_{B+}/\sigma_{B+}$ increases, due to conditioning on observed $Z_{F}>1.96$; that is, $P(Z_{F}>1.96)$ increases as $\mu_{B+}/\sigma_{B+}$ increases, thereby increasing the denominator in equation (4). We note that the contour lines curve for Rules 1 and 2, but for Rule 3, which is based on the interaction test, they are straight. The linear contours for Rule 3 do not hold if the estimands for the subgroups are non-independent, nor will they hold if data are binary or counts, see Appendix 1 for further details.

For this illustration, we set *L *=* *1 for Rule 1 and one-sided significance of the interaction to 0.1 for Rule 3. Changing these thresholds will result in a shift in the contour plots, whilst Rule 2 does not require specification of an additional parameter and is fixed. It is possible to closely align the three rules by choosing appropriate values for *L* and the interaction type I error if necessary.

### *4.3 Effect of correlation between estimands when* $\sigma_{B+}=\sigma_{B-},\;\pi =0.5$ *and* ***ρ** = 0.5*

Including additional covariates $X_{i}$ in the trial analysis in equation (1) induces correlation between the treatment effect estimands (and therefore the statistics) for the two subgroups. The bottom row of Figure 3 shows how the conditional power changes when there is moderate/strong correlation (ρ=0.5) between the treatment effect estimates, compared with no correlation in the top row. In all cases, the contours are closer together. Conditional power for Rule 1 changes only slightly since it relies largely on the absolute size of $\mu_{B}-/\sigma_{B-}$, whilst Rules 2 and 3 rely on both groups to a greater extent.

We note here that, if the two estimands are independent (*ρ* = 0) *and* arise from normally distributed data with common sampling variance in the two subgroups, then conditional and unconditional power for Rule 3 are identical, since $X=1.96-Z_{F}$ and $Y=\frac{{\hat{\mu }}_{B+}-{\hat{\mu }}_{B-}}{SD({\hat{\mu }}_{B+}-{\hat{\mu }}_{B-})}-z_{\alpha_{I}/2}$ in Rule 3 will also be independent. A brief proof is given in Appendix 1. However, for non-normal data or normal data with differential variance in the two subgroups or correlated $\mu_{B+}$ and $\mu_{B-}$, conditional and unconditional power for the interaction test will not be identical.

### 4.4 Effect of relative subgroup size when sampling variance is homoscedastic across subgroups and ***ρ** = 0.5*

**Figure 4.**
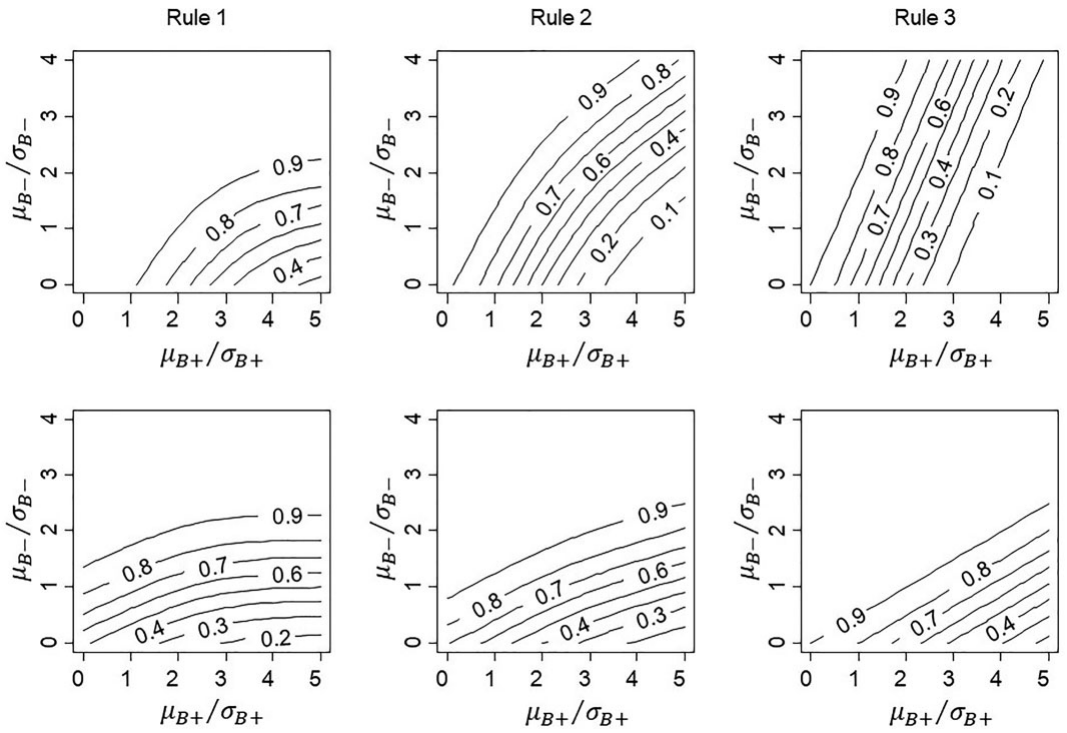
Conditional power for proposed rules with 20% of patients in subgroup B+ (top row) compared with 80% in B+ (bottom row), with *L *=* *1 for Rule 1 and one-sided significance of 10% for Rule 3.

We illustrate the influence of subgroup size on conditional power for the case where sampling variance is homoscedastic across biomarker subgroups. This will occur for normally distributed outcomes with the same sampling variance $\sigma^{2}$ in each subgroup, but does not necessarily hold for other distributions. Figure 4 shows how conditional power for each rule varied if either 20% or 80% of patients came from population B+. The proportion of patients sampled from the two sub-populations has a greater impact on conditional power than correlation between the two treatment effects.

If the trial sample contains a high proportion drawn from the B+ population (bottom row of Figure 4), then Rule 1 (with *L *=* *1) has lower power for small values of $\mu_{B+}/\sigma_{B+}$. This arises because we are conditioning on significance in the full population, and the B− patients contribute only a small amount to the overall. Conversely, because B− patients contribute a large amount to the overall analysis in the top row of Figure 4, the overall significance only occurs if there is good power that the observed $Z_{B-}$ exceeds *L* =1. Similar effects are observed for Rules 2 and 3, in that the overall-significance condition induces higher conditional power for approving treatment in B− at lower values of $\mu_{B+}/\sigma_{B+}$.

**Figure 5.**
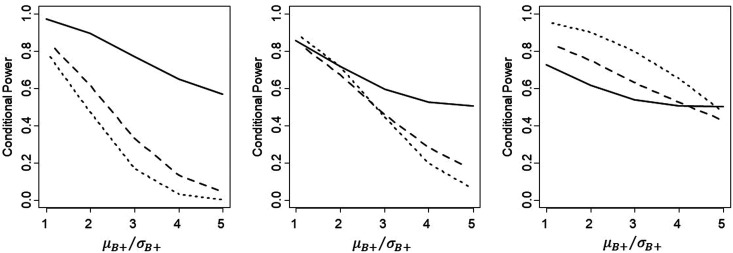
Comparison of conditional power for proposed Rule 1(solid line), Rule 2 (dashed line) and Rule 3 (dotted line), with the proportion of patients sampled from B+ equal to 20% (left panel), 50% (middle) and 80% (right panel), for *ρ* = 0 and with *L *=* *1 for Rule 1 and one-sided significance of 10% for Rule 3.

To provide further insight into the effect of relative sample size of the two subgroups, we plot conditional power against $\mu_{B+}/\sigma_{B+}$ for three different sampling proportions, 20:80, 50:50 and 80:20 with (*ρ* = 0) in Figure 5. This shows that, if the proportion of patients in this group is low (20%) or equal (50%), Rule 1 (with *L *=* *1) has highest conditional power across the plausible range of values of $\mu_{B+}/\sigma_{B+}$. Conversely, if the proportion of patients from B+ is high (80%), Rule 3 has uniformly highest conditional power. Rule 2 never has highest power in these scenarios, but we note that results are dependent on the chosen values of *L* for Rule 1 and the Type I error for interaction test, whilst Rule 2 has the advantage of not requiring additional parameters.

## 5 Illustrative applications

In order to illustrate how these rules might be used in practice, we retrospectively apply them to two completed phase III trials: a small cardiac surgery trial of 352 patients and equal size subgroups,^19^ and a much larger stroke trial (*n* = 7513) which evaluated betrixaban.^13^

### 5.1 AMAZE trial in cardiac surgery

AMAZE was a cardiac surgical trial in patients with atrial fibrillation (rapid/irregular heart rhythm).^19^ This multi-centre RCT randomised 352 patients 1:1 to a technique called ablation in addition to planned surgery, or to planned surgery alone (control arm). The primary outcome was *return to sinus rhythm* at one year post-surgery (binary). Although not part of the intervention, at the discretion of the operating surgeon 150/352 (π=42.6%) randomised patients had a section of the heart, called the left atrial appendage (LAA), removed during the procedure. This may be considered a more extensive procedure, conferring higher probability of a positive outcome. We define subgroups by whether the LAA was removed B+ or left intact B−. Selected results from the AMAZE trial are shown in Table 2.

**Table 2.** Treatment effect estimates and Z-statistics for patients with the LAA left intact (B−) or removed (B+) and overall in the AMAZE trial.

Patient Group	LAA left intact B−	LAA removed B+	Full population *F*
Estimand	${\hat{\beta }}_{1}$	${\hat{\beta }}_{1}+{\hat{\beta }}_{3}$	$\hat{\beta }$ across subgroups
Log odds ratio (standard error)	0.461 (0.378)	1.406 (0.472)	0.863 (0.320)
Z-statisic	1.220	2.981	2.697

We estimate conditional power for approval of ablation in B− assuming that actual trial results were our estimates of group-specific effects before the trial started. For Rule 1, there would 74% power to observe a treatment effect in B− if the threshold was set at *L *=* *1. Rule 2 is not met in AMAZE since $Z_{F}<Z_{B+}$; assuming observed trial results were “true”, conditional power was only 38%. Finally, the interaction Rule 3 was also just met if we set the decision threshold for the two-sided test statistic to 10% (interaction test *p *=* *0.119). Conditional power based on observed results was 37% for Rule 3; had this been specified during design of the trial, sample size could have been increased to increase confidence that Rule 3 would be met.

### 5.2 APEX trial in patients at high risk of stroke

Recall that one of the trials motivating this study compared treatment with the anticoagulant betrixaban with standard treatment of enoxaparin amongst hospitalised medically ill patients.^13^

The primary outcome was a composite of clinical events caused by blood clotting (deep vein thrombosis, non-fatal pulmonary embolism or death from thromboembolism) up to day 42 post-randomisation.

The planned analysis took a sequential testing approach, but rather than starting with the full trial population, the order of testing began with a subgroup with a high chance of treatment response (but smaller treatment effect), followed by testing in two other pre-specified, progressively inclusive cohorts as follows:

1. Compare treatment arms in patients with elevated D-dimer level (for illustration can be considered B−).
2. Compare treatment arms in patients with elevated D-dimer or age ≥75 (for illustration can be considered extended B−).
3. Compare treatment arms in all enrolled patients (full population, *n* = 7513).

If any test was negative, all subsequent tests were reported as exploratory. We provide selected results from the original trial publication in Table 3.

**Table 3.** Treatment effect estimates and Z-statistics for patients with the elevated d-dimer (B−) or non-elevated d-dimer (B+) and overall in the APEX trial.

Patient group	Elevated D-dimer B−	Non-elevated D-dimer B+	Full Population *F*
Events in treated patients	132/1914 (6.9%)	33/1198 (2.8%)	165/3112 (5.3%)
Events in control patients	166/1956 (8.5%)	67/1218 (5.5%)	223/3174 (7.0%)
Relative risk (95%CI)	0.81 (0.65, 1.00)	0.50 (0.33, 0.75)	0.76 (0.63, 0.92)
Log Relative Risk(Standard Error)	−0.21 (0.11)	−0.69 (0.21)	-0.28 (0.10)
Z-statistic	1.85	3.31	2.83

As the table shows, the first analysis including B− patients was not significant at the traditional threshold *p *=* *0.054, so that subsequent analyses were treated as exploratory, even though the experimental treatment effect was greater in the B+ subgroup and overall analyses. Adopting a sequential strategy, conditional on a significant effect of betrixaban in the full population, our proposed rules for the elevated B− subgroup would result in the following recommendations:

1. Rule 1: B− would be approved if a one standard error threshold (*L* = 1) was defined *a priori* as a clinically acceptable treatment effect. Assuming the trial result was “true” as for the previous example, the conditional power of this rule was 91%. If a significant effect in this subgroup was necessary (L≥1.96), as suggested by the original trial analysis, then Rule 1 was not met.
2. Rule 2 was also not met because group B− data resulted in a decrease in the Z-statistic for the full population compared to B+. This has arisen because B+ patients had a much higher treatment effect despite the lower event rate. The conditional power of this rule was only 34%.
3. There was a significant interaction between subgroup and treatment (one-sided *p* = 0.0184) so that this trial also fails Rule 3 – the conditional power was 43%.

In summary, using our proposed sequential testing procedure, betrixaban would be recommended for treatment in elevated d-dimer patients only if we were prepared to accept a lower treatment effect compared with non-elevated d-dimer patients and that lower treatment effect resulted in a Z-statistic of at most 1.85 standard errors ($Z_{B-}>1.85$).

### 5.3 Implications for trial design

In this context, investigators define and document decision rules for the primary trial analysis during the design stage. Our proposal is that, should a separate decision on approval of a subgroup be required, then a decision rule should be agreed in discussion with regulators or other appropriate decision makers during the design phase. Our evaluation of three potential rules illustrates how to investigate the efficiency of different rules, although parameter inputs will be specific to each trial and will depend on available information around potential efficacy.

Although our rules rely on *Z*-statistics for hypothesis tests, it is more usual to work with potential treatment effects and their standard deviations when designing a trial. Empirical estimates of variation in the primary outcome are typically available, particularly for the control arm of the trial. This may be a standard deviation for a continuous outcome, or the baseline risk of an event for patients receiving the current best treatment. Given these estimates, the sample size required for an overall significant treatment effect, the proposed sampling proportion, and the Rule 1 threshold *L* can be decided to ensure that the treatment effect in subgroup B− lies above a minimum treatment effect. In a similar way, the Rule 3 significance level can be chosen to ensure there is sufficient power to find an interaction if the B− estimate is much lower than B+.

The stages of design are as follows:

1. Using initial estimates of design parameters, including the sampling proportion *π* and correlation *ρ*, calculate the power of the test for the expected value of the treatment effect in the full trial population.
2. Choose a decision rule for recommending treatment in B− based on considerations of clinically important treatment effects, safety and biological mechanisms.
3. Given the sampling proportion *π* and the expected treatment effect sizes in the two sub-populations, calculate the power of your preferred rule, conditional on a significant overall test.
4. Calculate the power of the sequential testing strategy as the product of conditional and unconditional power in 1 and 3.

In practice the final power calculations will require an iterative process between calculation and elicitation of expert clinical knowledge of treatment effects and associated variance components, finalised in discussion with regulators or other decision makers.

## 6 Discussion

### 6.1 Overview of results

Frequentist rules to assess whether approval of a new treatment should be accepted in a lower response subgroup, conditional on a significant effect overall, have been developed and evaluated. Approval based solely on a significant overall test may be unacceptable if there are severe side effects and/or if the subgroup drawn from the low response population is under-represented due to enrichment sampling. Rules are based either on measures of influence, such as the size of the effect in this subgroup, or the increase in significance due to inclusion of the subgroup, or on the difference in effect size between the groups (interaction). When choosing a rule during trial design, as well as specifying estimates of the expected outcomes and their variance components, investigators must either take a random sample from the full population, in which case the trial will represent clinical practice, or decide the proportion of patients to be sampled from each sub-population. Using conditional power as a measure of efficiency, the proportion of patients drawn from each sub-population had a large impact, but correlation between the groups induced by covariate adjustment was less important. For all rules, conditional power decreased as μ_*B*_+__/σ_*B*_+__ increased for fixed μ_*B*_−__/σ_*B*_−__.

### 6.2 Discussion of individual rules

After ensuring that $Z_{F}>1.96$, the simplest approach is to perform tests in B+ and B− separately as part of a closed testing procedure, and allow a more relaxed significance level for $Z_{B-}$ (Rule 1). This significance level is related to both the proportion of the trial sample in B− and the effect size that is acceptable given the safety profile of the treatment. Hence, the level can be set based on prior knowledge of treatment effect and prevalence of low responders in the population. In our illustration, for trials with ≤50% of trial patients in B+, Rule 1 had the highest power for our chosen threshold of $Z_{B-}>1$.

Rule 2 (B− patients should increase significance) is rather *ad hoc* but has the benefit of not requiring specification of an additional parameter. To satisfy Rule 2, B− must preserve at least some proportion of the estimated efficacy of B+. Further, the conditional power of Rule 2 decreases as the proportion of patients from B− decreases, which is also an attractive property. Despite these benefits, Rule 2 never demonstrated highest conditional power in our analyses.

Rule 3 uses an interaction test with a relaxed significance level to recommend approval in the B− sub-population. Interaction tests in clinical trial publications typically aim to identify heterogeneity between subgroups and are mainly purely exploratory. However, a significant subgroup-treatment interaction at the 5% level may not preclude approval in the lower response group, provided that there is a minimum level of efficacy, particularly if side effects are mild or there are few alternatives for this subgroup. Our more targeted objective is equivalent to testing whether the difference in treatment effects between the two groups is within acceptable limits and can be reconstructed as an equivalence or non-inferiority test. That is, the (interaction test) significance level can be based on *a priori* estimates of the maximum acceptable difference between the two subgroups ($\equiv \beta_{3}$ in equation 1). In our analyses, for trials where >50% of patients arise from B+, Rule 3 had the highest power to approve B− given an overall significant result.

### 6.3 Regulator input

In practice, acceptability of these approaches will depend on regulators (for drug trials) or commissioners (for academic trials). Since the conditional power of the rules depends crucially on the values chosen for the parameters *L* and *α_I_*, as well as patient sampling, prevalence of high/low responders and analysis methods, early engagement with regulators/commissioners to discuss these decision rules is worthwhile. Discussions also need to consider potential harms (side effects), in order to set realistic and acceptable targets for efficacy. In practice, investigators/sponsors will be required to pre-specify and document these decision rules in discussion with regulators.

### 6.4 Strength, weaknesses and future research

One benefit of our proposed decision rules is that closed-form expressions for conditional power are available for continuous, binary and count outcomes (assuming known variances). This makes estimation of sample sizes relatively simple, and a wide range of scenarios can be explored during the design phase.

In our examples, we used retrospective power calculations to show the differences in conditional power for the three rules based on trial results. We stress that these calculations were provided for illustration only and we do not endorse retrospective power calculations to aid interpretation of statistically non-significant trial results (see for example Hoenig and Heisey^20^).

In common with many statistical methods, there is an underlying assumption of normality when using generalised linear models. This will hold for most adequately powered, phase III trials where analysis is completed on a scale for which the sampling distributions of estimated coefficients can be assumed normal (e.g. logistic, log). For small trials, or for estimands with very skewed distributions, asymptotic approximations may not hold and analyses should be checked using simulations.

In this paper, we provided expressions for the case where patients were randomised 1:1 to the experimental and control arms, although extension to other allocation ratios is straightforward. It would also be relatively straightforward to extend the methods to biomarkers with more than two levels, although the number of patients at each level is likely to be small in this case, resulting in low conditional power for all proposed rules. An exception might be for biomarkers with ordered levels, in which case the subgroup effect and interaction with treatment could be linear terms in the analysis (equation (1)).

For time-to-event outcomes, power of the study depends directly on the number of events occurring rather than on the number of patients, so that power would also depend on recruitment and censoring patterns. Methods would need to be extended to accommodate these features. Further, we have not embedded these results in more formal decision analytic methods, and this would require further specification of costs, harms (side effects) and utilities (benefits) and would depend on the perspective of the investigator (sponsor or health provider).

In summary, in situations where additional conditions are required for approval of a new treatment in a lower response subgroup, easily applied rules based on minimum effect sizes and relaxed interaction tests are available. These depend on trial design characteristics, particularly the proportion of patients sampled from the two subgroups and must be pre-specified and documented in the *Statistical Analysis Plan*.

## Supplemental Material

sj-zip-1-smm-10.1177_09622802211017574 - Supplemental material for Frequentist rules for regulatory approval of subgroups in phase III trials: A fresh look at an old problem Click here for additional data file. Supplemental material, sj-zip-1-smm-10.1177_09622802211017574 for Frequentist rules for regulatory approval of subgroups in phase III trials: A fresh look at an old problem by K Edgar, D Jackson, K Rhodes, T Duffy, C-F Burman and LD Sharples in Statistical Methods in Medical Research

## Appendix 1. Conditional power calculations

The following shows how to calculate the conditional power for each rule, given pre-specified parameters $\mu_{B+},\mu_{B-},\sigma_{B+},\sigma_{B-}$, the proportion of patients in B+ (*π*), and the correlation between the subgroup treatment effects (and therefore the subgroup test statistics $Z_{B+}$ and $Z_{B-}$) *ρ*.

Given pre-specified treatment effects $\mu_{B+}$ and $\mu_{B-}$ for the subgroups and planned sampling proportion from the B+ sub-population *π*, the full population treatment effect is

$$\mu_{F}=\pi \mu_{B+}+(1-\pi )\mu_{B-}$$

We have that the conditional power for each rule is

$$P(Rn|Z_{F}>1.96)=\frac{P(Rn,Z_{F}>1.96)}{P(Z_{F}>1.96)}$$

where, for variance components $\sigma_{B+}^{2}$ and $\sigma_{B-}^{2}$ and correlation *ρ*, we have

$$Z_{F}=\frac{{\hat{\mu }}_{F}}{\sigma_{F}}=\frac{\pi \sigma_{B+}Z_{B+}+(1-\pi )\sigma_{B-}Z_{B-}}{\sqrt{\pi^{2}\sigma_{B+}^{2}+{\left(1-\pi \right)}^{2}\sigma_{B-}^{2}+2\pi (1-\pi )\rho \sigma_{B+}\sigma_{B-}}}\;$$

The denominator does not depend on the form of any proposed rule and is given by

$$P(Z_{F}>1.96)=\Phi (\mu_{F}/\sigma_{F}-1.96)$$

Note that the right-hand side is a function of three location parameters $\mu_{B+},\;\mu_{B-}$ and *π*, and three variance parameters $\sigma_{B+},\;\sigma_{B-}$, *ρ* (see equations (2) and (3)), and conditional on these the standard normal deviate can be obtained from any statistical software.

**A.1 Rule 1 numerator**

For rule 1, the numerator of the conditional power is given by the expression

$$P(Z_{B-}>L,Z_{F}>1.96)$$

We make the transformation $X=1.96-Z_{F}$ and $Y=L-Z_{B-}$, and find their distribution as follows

$$\begin{array}{l} E(X)=E(1.96-Z_{F})=1.96-\mu_{F}/\sigma_{F}\\ E(Y)=E(L-Z_{B-})=L-\mu_{B-}/\sigma_{B-} \end{array}$$

Because *Z_F_* and $Z_{B-}$ are *Z* test statistics (i.e. assumed  N(0,1)), the variances of X and Y are given by

$$\begin{array}{l} Var(X)=\mathrm{Var}(1.96-Z_{F})=\mathrm{Var}(-Z_{F})=1\\ Var(Y)=\mathrm{Var}(L-Z_{B-})=\mathrm{Var}(-Z_{B-})=1 \end{array}$$

The covariance of X and Y is

$$\begin{array}{l} Cov(X,Y)=\mathrm{Cov}(1.96-Z_{F},L-Z_{B-})\\ =\mathrm{Cov}(-(\frac{\pi \sigma_{B+}Z_{B+}+(1-\pi )\sigma_{B-}Z_{B-}}{\sigma_{F}}),-Z_{B-})\\ =\frac{\pi \sigma_{B+}}{\sigma_{F}}Cov(Z_{B+},Z_{B-})+\frac{(1-\pi )\sigma_{B-}}{\sigma_{F}}Cov(Z_{B-},Z_{B-})\\ =\frac{\pi \rho \sigma_{B+}}{\sigma_{F}}+\frac{(1-\pi )\sigma_{B-}}{\sigma_{F}}=\frac{\pi \rho \sigma_{B+}+(1-\pi )\sigma_{B-}}{\sigma_{F}} \end{array}$$

Therefore, the joint distribution of X and Y is

$$\left(\begin{array}{l} X\\ Y \end{array}\right)∼\mathrm{BVN}\left(\left(\begin{array}{l} 1.96-\mu_{F}/\sigma_{F}\\ L-\mu_{B-}/\sigma_{B-} \end{array}\right),\left(\begin{array}{ll} 1 & \frac{\pi \rho \sigma_{B+}+(1-\pi )\sigma_{B-}}{\sigma_{F}}\\ \frac{\pi \rho \sigma_{B+}+(1-\pi )\sigma_{B-}}{\sigma_{F}} & 1 \end{array}\right)\right)$$

The numerator is P(X≤0,Y≤0) and can be obtained from standard statistical software.

**A.2 Rule 2 numerator**

The numerator for Rule 2 is given by $P(Z_{F}>Z_{B+},Z_{F}>1.96)$. Making the transformations, $X=1.96-Z_{F}$ and $Y=Z_{B+}-Z_{F}$, the joint distribution of X and Y is found as follows:

X is the same as for Rule 2, so that the expectation and variance of X are again $1.96-\mu_{F}/\sigma_{F}$ and 1, respectively.

The expectation and variance of Y are

$$\begin{array}{l} E(Y)=E(Z_{B+}-Z_{F})=\mu_{B+}/\sigma_{B+}-\mu_{F}/\sigma_{F}\\ Var(Y)=\mathrm{Var}(Z_{B+}-Z_{F})=\mathrm{Var}(Z_{B+})+\mathrm{Var}(Z_{F})-2Cov(Z_{B+},Z_{F})\\ =2-2\times \mathrm{Cov}(\frac{\pi \sigma_{B+}Z_{B+}+(1-\pi )\sigma_{B-}Z_{B-}}{\sigma_{F}},Z_{B+})\\ =2-2\times \frac{\pi \sigma_{B+}Var(Z_{B+})+(1-\pi )\sigma_{B-}Cov(Z_{B+},Z_{B-})}{\sigma_{F}}\\ =2\times (1-\frac{\pi \sigma_{B+}+\rho (1-\pi )\sigma_{B-}}{\sigma_{F}}) \end{array}$$

The covariance of X and Y is

$$\mathrm{Cov}(X,Y)=\mathrm{Cov}(1.96-Z_{F},Z_{B+}-Z_{F})=\mathrm{Var}(Z_{F})-\mathrm{Cov}(Z_{F},Z_{B+})=1-\frac{\pi \sigma_{B+}+\rho (1-\pi )\sigma_{B-}}{\sigma_{F}}$$

The joint distribution of X and Y for Rule 2 is

$$\left(\begin{array}{l} X\\ Y \end{array}\right)∼\mathrm{BVN}\left(\left(\begin{array}{l} 1.96-\mu_{F}/\sigma_{F}\\ \mu_{B+}/\sigma_{B+}-\mu_{F}/\sigma_{F} \end{array}\right),\left(\begin{array}{ll} 1 & 1-\frac{\pi \sigma_{B+}+\rho (1-\pi )\sigma_{B-}}{\sigma_{F}}\\ 1-\frac{\pi \sigma_{B+}+\rho (1-\pi )\sigma_{B-}}{\sigma_{F}} & 2\times (1-\frac{\pi \sigma_{B+}+\rho (1-\pi )\sigma_{B-}}{\sigma_{F}}) \end{array}\right)\right)$$

Again, the numerator for conditional power is P(X≤0,Y≤0), which we obtain from standard statistical software.

**A.3 Rule 3 numerator**

For this rule the numerator is $P(({\hat{\mu }}_{B+}-{\hat{\mu }}_{B-})/SD({\hat{\mu }}_{B+}-{\hat{\mu }}_{B-})<z_{\alpha_{I}/2},Z_{F}>1.96)$, where $z_{\alpha_{I}/2}$ is the $100(1-\alpha_{I})\%$ quantile from the standard normal distribution.

We make the transformations, $X=1.96-Z_{F}$ and $Y=\frac{{\hat{\mu }}_{B+}-{\hat{\mu }}_{B-}}{\sigma_{3}}-z_{\alpha_{I}/2}$, where $\sigma_{3}^{2}=\sigma_{B+}^{2}+\sigma_{B-}^{2}-2\rho \sigma_{B+}\sigma_{B-}$.

Again the expectation and variance of X are $1.96-\mu_{F}/\sigma_{F}$ and 1, respectively.

The expectation and variance of Y are

$$\begin{array}{l} E(Y)=E(\frac{{\hat{\mu }}_{B+}-{\hat{\mu }}_{B-}}{SD({\hat{\mu }}_{B+}-{\hat{\mu }}_{B-})}-z_{\alpha_{I}/2})=\frac{\mu_{B+}-\mu_{B-}}{\sigma_{3}}-z_{\alpha_{I}/2}\\ Var(Y)=\mathrm{Var}(\frac{{\hat{\mu }}_{B+}-{\hat{\mu }}_{B-}}{SD({\hat{\mu }}_{B+}-{\hat{\mu }}_{B-})}-z_{\alpha_{I}/2})=\frac{\mathrm{Var}({\hat{\mu }}_{B+}-{\hat{\mu }}_{B-})}{\mathrm{Var}({\hat{\mu }}_{B+}-{\hat{\mu }}_{B-})}=1 \end{array}$$

The covariance of X and Y is given by

$$\begin{array}{l} Cov(X,Y)=\mathrm{Cov}(1.96-(\frac{\pi \sigma_{B+}Z_{B+}+(1-\pi )\sigma_{B-}Z_{B-}}{\sigma_{F}}),\frac{{\hat{\mu }}_{B+}-{\hat{\mu }}_{B-}}{\sigma_{3}}-z_{\alpha_{I}/2})\\ =\frac{\mathrm{Cov}(-\pi \sigma_{B+}Z_{B+}-(1-\pi )\sigma_{B-}Z_{B-},\sigma_{B+}Z_{B+}-\sigma_{B-}Z_{B-})}{\sigma_{F}\sigma_{3}}\\ =\frac{-\pi \sigma_{B+}^{2}Var(Z_{B+})+\pi \sigma_{B+}\sigma_{B-}Cov(Z_{B+},Z_{B-})-(1-\pi )\sigma_{B-}\sigma_{B+}Cov(Z_{B+},Z_{B-})+(1-\pi )\sigma_{B-}^{2}Var(Z_{B-})}{\sigma_{F}\sigma_{3}}\\ =\frac{\rho (2\pi -1)\sigma_{B+}\sigma_{B-}-\pi \sigma_{B+}^{2}+(1-\pi )\sigma_{B-}^{2}}{\sigma_{F}\sigma_{3}} \end{array}$$

Then the joint distribution of X and Y is

$$\left(\begin{array}{l} X\\ Y \end{array}\right)∼\mathrm{BVN}\left(\left(\begin{array}{l} 1.96-\mu_{F}/\sigma_{F}\\ \frac{\mu_{B+}-\mu_{B-}}{\sigma_{3}}-z_{\alpha_{I}/2} \end{array}\right),\left(\begin{array}{ll} 1 & \frac{\rho (2\pi -1)\sigma_{B+}\sigma_{B-}-\pi \sigma_{B+}^{2}+(1-\pi )\sigma_{B-}^{2}}{\sigma_{F}\sigma_{3}}\\ \frac{\rho (2\pi -1)\sigma_{B+}\sigma_{B-}-\pi \sigma_{B+}^{2}+(1-\pi )\sigma_{B-}^{2}}{\sigma_{F}\sigma_{3}} & 1 \end{array}\right)\right)$$

Again, the numerator for conditional power is P(X≤0,Y≤0), which we obtain from standard statistical software.

**A.4 Equality of conditional and unconditional power for rule 3 when *ρ* = 0, data are normally distributed and subgroups have the same sampling variance**

To explore when conditional and unconditional power are the same, we identify conditions when $P(R3|Z_{F}>1.96)=P(R3)$, or equivalently, the correlation of *R*3 and $Z_{F}>1.96$ is zero.

For Rule 3, conditional power is defined as $P(({\hat{\mu }}_{B+}-{\hat{\mu }}_{B-})/SD({\hat{\mu }}_{B+}-{\hat{\mu }}_{B-})<z_{\alpha_{I}/2}|Z_{F}>1.96)$ and we made the transformations, $X=1.96-Z_{F}$ and $Y=\frac{{\hat{\mu }}_{B+}-{\hat{\mu }}_{B-}}{\sigma_{3}}-z_{\alpha_{I}/2}$, so that the conditional power can be written $P(Y<z_{\alpha_{I}/2}|X>1.96)$.

Recall that the covariance of X and Y is

$$\frac{\rho (2\pi -1)\sigma_{B+}\sigma_{B-}-\pi \sigma_{B+}^{2}+(1-\pi )\sigma_{B-}^{2}}{\sigma_{F}\sigma_{3}}$$

If there is no correlation between the two subgroup treatment estimates *ρ* = 0, then this will become

(6) $$\frac{-\pi \sigma_{B+}^{2}+(1-\pi )\sigma_{B-}^{2}}{\sigma_{F}\sigma_{3}}$$

Recall that for the normal distribution case with *n* patients allocated to each treatment arm and common sampling variance $\sigma^{2}$ in the two subgroups, $\sigma_{B+}^{2}=2\sigma^{2}/\pi n$ and $\sigma_{B-}^{2}=2\sigma^{2}/(1-\pi )n$. Substituting these into equation (6) results in zero correlation between Rule 3 and the overall significance condition, and shows that the interaction rule is independent of the condition for this case.

For binary responses and usng a logit link, $\sigma_{B+}^{2}=[1/(\theta_{1B+}(1-\theta_{1B+}))+1/(\theta_{0B+}(1-\theta_{0B+})]/\pi n$ and $\sigma_{B-}^{2}=[1/(\theta_{1B-}(1-\theta_{1B-}))+1/(\theta_{0B-}(1-\theta_{0B-})]/((1-\pi )n)$ where *θ_jk_* is the probability of an event in treatment arm *j* and subgroup *k*. In this case, the covariance is zero only if

$$1/(\theta_{1B+}(1-\theta_{1B+}))+1/(\theta_{0B+}(1-\theta_{0B+})=1/(\theta_{1B-}(1-\theta_{1B-}))+1/(\theta_{0B-}(1-\theta_{0B-})$$

In general, this will not hold. A similar situation applies for count data.

In summary, if the two subgroup estimates are correlated (ρ≠0 due to covariate adjustment) or sampling variance in the two groups differ, either because the data are not normally distributed or because outcome measurements in the two groups have different variances, then conditional and unconditional power are not necessarily equivalent under Rule 3.
